# High-resolution thermal infrared dataset for airborne person detection in SAR missions

**DOI:** 10.1038/s41597-026-07663-9

**Published:** 2026-07-07

**Authors:** Johannes Büttner, Kilian Führer, Andreas Fritz, Jonathan Zender, Bernd R. Pinzer

**Affiliations:** Insitute for Machine Vision, University of Applied Sciences Kempten, Bahnhofstraße 61, 87435 Kempten, Germany

## Abstract

The utilization of uncrewed aerial vehicles (UAVs) in search and rescue (SAR) operations has become increasingly prevalent because the deployment of UAVs is expected to facilitate a higher degree of operational flexibility while simultaneously reducing costs. Currently, commercially available UAVs can be equipped with low-resolution thermal infrared (IR) cameras with typical resolutions of 640 × 512 pixels, which generally are evaluated manually by the SAR teams during an operation. Automatic person detection in IR images still remains a challenge. The objective of the proposed AIResQ dataset is to significantly enhance the performance of object detectors in the IR domain, employed in SAR operations for missing and potentially injured persons. AIResQ comprises 9,788 IR images with a resolution of up to 2048 × 1536 pixels captured from drone perspectives with a handheld camera under varying weather conditions and in different terrains. Additionally, AIResQ displays persons in atypical poses. In order to test new object detectors in the context of SAR, we established a benchmark dataset stemming from exercises with real drone flights together with SAR organizations.

## Background & Summary

### Background

Uncrewed aerial vehicles (UAVs) are extremely helpful for wilderness search and rescue (SAR) missions^1^, where the condition and position of missing persons are unknown and the highest priority is to locate the persons. The advantages of UAVs for wilderness search include the ability to cover large areas and to capture images automatically, even in inaccessible zones where searching by humans would be too dangerous. The relatively low operational cost and straightforward handling characteristics compared to helicopters, the possibility to combine a swarm of drones in order to use formation flights for searching^2^, and automatic mission planning involving lost person behaviour models to maximize the probability for finding persons^3^ are additional advantages.

Person detection in the visible spectrum, i.e. on 3-channel red/green/blue (RGB) images, has a long history. In the SAR context, automatic person recognition in drone video footage was already discussed two decades ago^4^. More recently, Sambolek and Ivasic-Kos^1,5^ evaluated CNN-based object detectors and their performance after transfer learning. From a practical point of view, a recent case study from an SAR mission in Japan^6^ described in detail the search for a lost hiker, where large numbers of drone images were automatically evaluated by two CNN models that had shown promising results in the lab. However, the report concludes that none of the models reliably detected persons, but suffered from both false positives (tree trunks and rocks classified as humans) and false negatives (SAR staff known to be in the area and visible to the human eye were not detected). The models were chosen among 19 models that were trained on the three prevailing representative SAR datasets in the RGB modality, including YOLO^7^, SSD^8^, FasterRCNN^9^, RetinaNet^10^, EfficientDet^11^. Due to the unsatisfactory results in a real wilderness search mission, the authors urge that more datasets and more diverse datasets for SAR missions should be created, along with model improvements and standardization of model evaluation. The challenges in airborne whole body detection are high altitude, large angles of observation, and wilderness scenes^12^. Person detection in infrared images draws from the results in RGB detectors, but suffers from lower resolution and a pronounced domain gap. In 2005, Davis and Keck^13^ used correlation based matching of a generalized template, followed by an AdaBoosted ensemble classifier. Hot spot detection and classification based on a DCT-descriptor was used by Teutsch *et al*. in^14^. The classification of a hot spot was achieved at that time by feature extraction followed by a classifier algorithm^14^. Herrmann *et al*.^15^ were the first to use a CNN for the second step, the hot spot classification, in order to find persons in IR images. In 2018, Herrmann *et al*.^16^ used pretrained (on RGB images) CNN detectors and domain adaptation to transfer existing models to the IR domain. Subsequently, typical configurations of RGB-detectors like YOLO, SSD, FasterRCNN were applied for transfer learning in the IR domain^17–22^. Huda *et al*.^23^ had a closer look at the influence of the transfer learning dataset, and trained a YOLOv3 network with different combinations of selected images. There was no clear indication of what the most important influence was.

### Related Work

Compared to the RGB domain, there is not as much specific training data available in the IR domain. Several publications use their own custom dataset^19,24,25^, but these are not publicly available. Public IR datasets relevant for person detection have been summarized by Huda *et al*.^23^. Since then, a total of eight datasets have been published which are fitting with the purpose of identifying persons in IR images: WiSARD^26^, HIT-UAV^27^, VTUAV^28^, CART^29^, BIRDSAI^30^, RGBTDronePerson^31^, NII-CU MAPD^32^ and POP^33^. These datasets include labels for the individual classes and vary in terms of the recording environment, number of images and number of bounding boxes. The following Table 1 presents a synopsis of the pertinent data, including resolution, quantity of images and bounding boxes, and the surrounding environment of these datasets as well as the AIResQ^34^ and Benchmark^35^ datasets described in this contribution. The VTUAV and CART dataset were excluded from further analysis because of unsuitable labels, which could not be transformed into bounding box format.

**Table 1:** Overview of related datasets and AIResQ dataset with comparison of resolution, image count, number of bounding boxes with label “person” and surrounding environment while recording.

Dataset	Resolution [px]	Images	Bounding Boxes	Environment
**Benchmark (ours)**	640 × 512	1,988	2,643	Wilderness
**WiSARD**	640 × 512	28,261	27,544	Wilderness
**HIT-UAV**	640 × 512	2,866	12,312	Urban
VTUAV^1^	1920 × 1080	833,274	—	Urban
CART^2^	640 × 512	3,076	—	Wilderness
**BIRDSAI**	698 × 519 & 640 × 512	61,994	12,531	Wilderness
**RGBTDronePerson**	640 × 512	6,125	60,258	Wilderness
**NII-CU MAPD**	640 × 512	9,018	23,037	Wilderness
**POP**	1280 × 1024	8,767	26,811	Wilderness
**AIResQ(ours)**	2048 × 1536 & 1024 × 768	9,788	17,550	Wilderness

^1^Number of IR images only. VTUAV is suitable for object tracking tasks, not for SAR tasks, as there is little variance in the scenes, and labels are segmentation masks.

^2^Label format is in segmentation masks and not in bounding boxes.

### Motivation

Why do we need yet another IR dataset? Working together with SAR organizations, we realized that none of the published images matched the specific requirements of real life SAR missions carried out in the alpine region: non-urban environment with trees and rocks, unusual poses and drone perspective. In scenarios involving animals or arid, stony terrain during high-temperature summer days, environmental surfaces often reach or exceed human body temperature. Consequently, thermal intensity alone is insufficient for reliable discrimination. High-resolution spatial data is therefore essential to distinguish human morphological contours from environmental heat sources and non-human objects. Most importantly, there is no high-resolution dataset available, anticipating the technological advancement of IR cameras attached to UAVs. Therefore, this paper contributes threefold to the active research field of IR person detection in SAR missions: 1) We collected a new training dataset, called AIResQ^34^, filling the gaps that currently available IR datasets leave for SAR missions, most notably high resolution for training detectors for the next generation of drone IR cameras, alpine landscapes, and simulating accidents of the depicted persons via a handheld camera. 2) We established a benchmark dataset, collected from simulated search missions together with SAR organizations via drone flights. The benchmark dataset reflects the requirements and scene characteristics of real search missions, as the drones were flown by experienced SAR staff and the dataset has been assembled for the purpose of validating the trained object detectors as a comparable benchmark. 3) We show that existing datasets are not well suited to achieve robust detection with high accuracy in our benchmark dataset. Using the AIResQ^34^ dataset, however, the object detection models increase their detection performances on our benchmark dataset.

### Data Overview

**Fig. 1:**
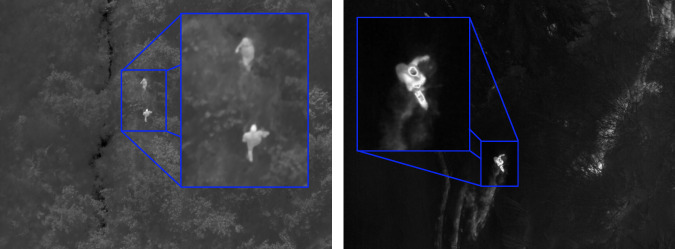
Example picture of AIResQ dataset in summer and winter: (Left) Two persons are depicted walking within a forest during the summer months, (Right) whilst a person is illustrated lying on a mountainside in a wintry environment, in a manner that is suggestive of a skiing accident.

AIResQ^34^ is a thermal images dataset with a resolution of 2048 × 1536 (6,506 images / 66,5%) and 1024 × 768 pixel (3,282 images / 33,5%). The dataset comprises 9,788 thermal images and 17,550 bounding boxes with persons in different scenarios. The whole dataset includes images from a full annual cycle, on different times of day, within several terrains and different weather patterns. The number of individuals visible in each image varies between zero and fifteen. Figure 1 illustrates two examples of images included in the dataset. The images depict a varying number of individuals in a range of postures, which are typical of those suffering injuries in an alpine setting in different temperature settings. The of images without human targets account for 28,5% of the total dataset.

To ensure a robust and comparative evaluation across all examined datasets, we supplemented our primary data collection with a dedicated benchmark gathered in collaboration with professional emergency response organizations, such as the Mountain Rescue Service. This additional benchmark serves as a unified ground truth reference, enabling a standardized performance analysis across diverse data sources. By integrating high-fidelity data from real-world rescue scenarios, we provide a consistent metric to validate the generalizability and reliability of our findings within the specific constraints of emergency operations.

## Methods

The objective is to generate a high-resolution infrared dataset that exhibits a markedly higher level of resolution in comparison to available datasets. In order to achieve this objective, the only commercially available infrared camera that can capture infrared images of up to three megapixels was utilized. Consequently, the scope of our recording endeavors has been constrained to a variety of elevated structures, including mountain cable cars, masts, tall towers and suspension and highway bridges. Only persons were labeled in the data annotation process. The dataset creation pipeline is comprised of three distinct stages: data capture, data preprocessing and data annotation.

### Data Collection

**Fig. 2:**
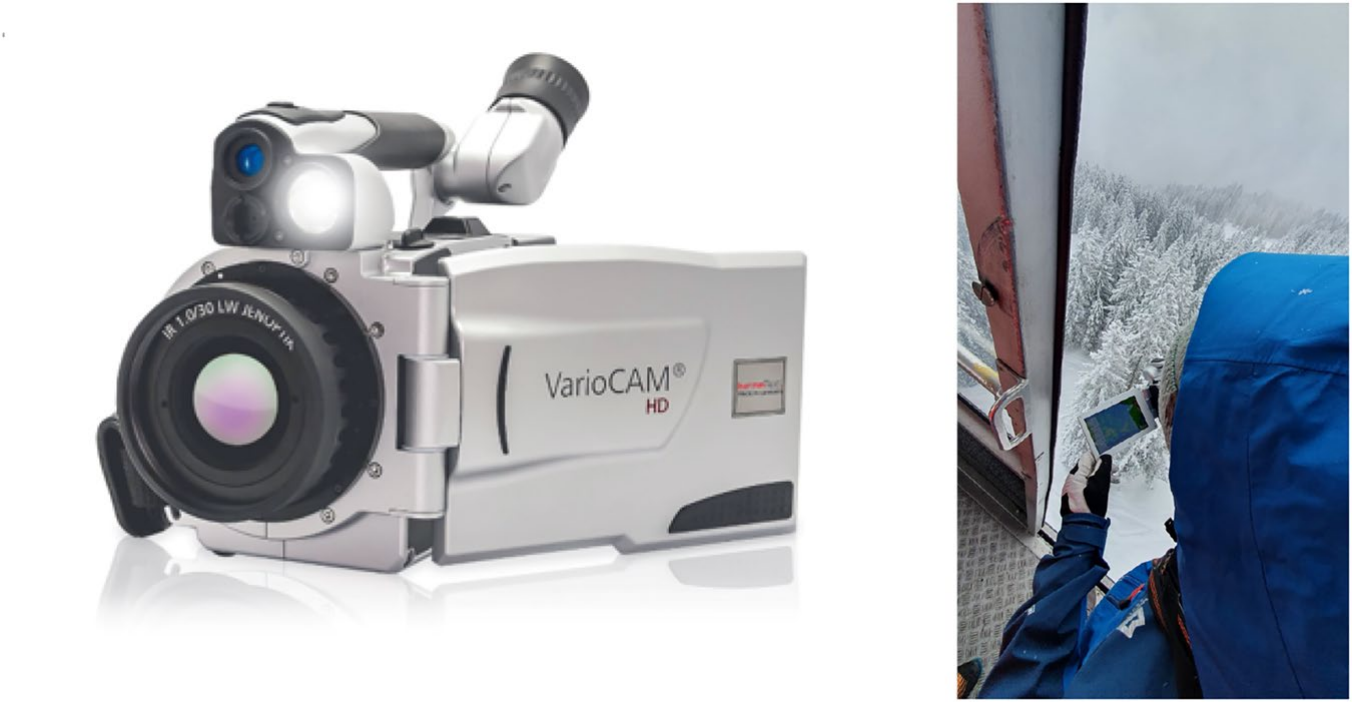
The camera utilized for data collection was a VarioCAM^®^ HD research. This camera is capable of capturing thermal images with a resolution of up to 2048  × 1536 pixels at a frequency of one image per ten seconds, within the long-wave infrared (LWIR^46^) range.

The data collection process was conducted using a VarioCAM^®^ HD research^36^ camera which is depicted in Fig. 2. At the completion of the data collection process 17,550 person labels from higher data acquisition locations were collected. The thermal imaging camera has a resolution of up to 2048 × 1536 pixels; however at this resolution, the recording frequency is reduced to one image per ten seconds. Consequently, this yielded a pure recording time of 9,788 images exceeding over 27 hours. Additionally, the camera - displayed in Fig. 2 - lacks remote triggering capabilities and had to be operated manually, thus precluding the possibility of combining it with a drone. Consequently, the images were predominantly obtained from the hatches of cabins of mountain cable cars, masts, towers, or bridges. Individuals discernible in the images were either bystanders or volunteers traversing the field area. The AIResQ^34^ dataset was recorded in a series of repetitive cycles in different locations.

**Fig. 3:**
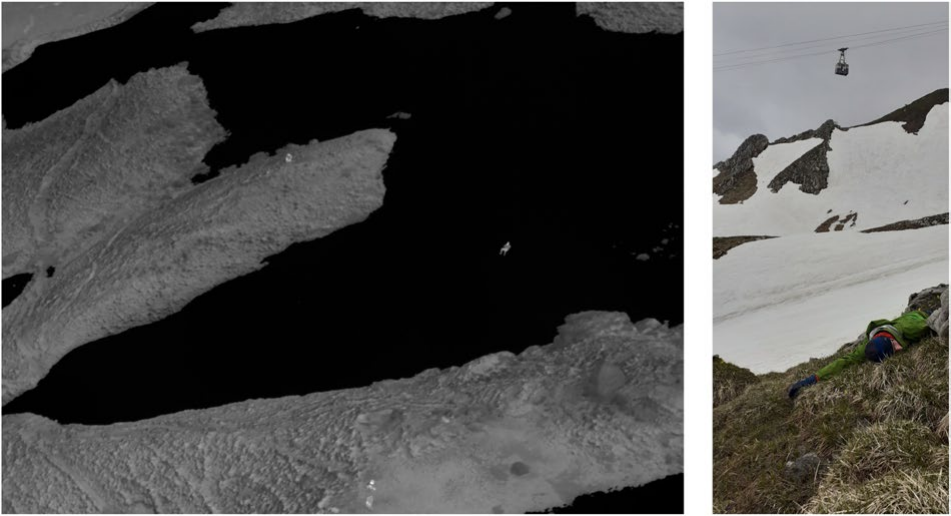
All pictures forming the AIResQ dataset were captured manually with a VarioCAM^®^ HD research from mountain cable cars, on towers and bridges. In order to simulate a range of poses typical of those assumed by injured individuals, the subjects were positioned in close proximity to the recording location. (Left) A view of the Nebelhorn mountain captured from a mountain cable car. (Right) An actor performing a casualty simulation, portraying an injured individual.

The thermal images’ drone-like perspective was achieved by capturing images from mountain cable cars, bridges, and observation towers, as shown in Fig. 3. The typical shooting height was found to be between 50 and 120 meters above ground level. This required the camera operator to be in an optimal position relative to the actors pretending injury. The dataset was collected with the help of volunteers, which placed themselves in unusual postures in the field of view of the camera operator. Prior to the publication of the dataset, all volunteers were requested to provide consent, and all volunteers consented to the publication. The creation process involved a cohort of voluntary participants ranging in age from 6 to 50 years, with a demographic distribution that was predominantly male. To ensure the precision of the captured scenarios, the poses of these primary actors were coordinated in real-time via radio communication. Furthermore, to enhance the validity of the dataset, we also recorded unscripted bystanders who were present in the mountain environment during the sessions. This combination of coordinated maneuvers and spontaneous pedestrian activity provides a comprehensive representation of both targeted rescue actions and naturalistic background movement.

The captured dataset is highly representative of real-world aerial thermal imaging, making it suitable for training robust object detection models. By accounting for sensor-specific characteristics - specifically high acquisition times of 130 to 150 milliseconds - the images exhibit the motion blur typically encountered in dynamic drone-based infrared acquisition. In order to demonstrate the suitability of AIResQ^34^ to train person detectors useful for SAR missions, we tested the resulting models against the benchmark dataset, which draws from a different distribution. Furthermore, the dataset encompasses a wide range of meteorological conditions reflective of the challenging environments found in SAR operations. These diverse weather and surface parameters are systematically documented in an accompanying JSON metadata file, providing a comprehensive basis for evaluating detector performance across varying atmospheric constraints. Additionally, the Technical Validation section provides a detailed analysis of bounding box dimensions, demonstrating that our dataset exhibits size distributions closely aligned with standard aerial benchmarks, further confirming its practical applicability.

### Data Preprocessing

**Fig. 4:**
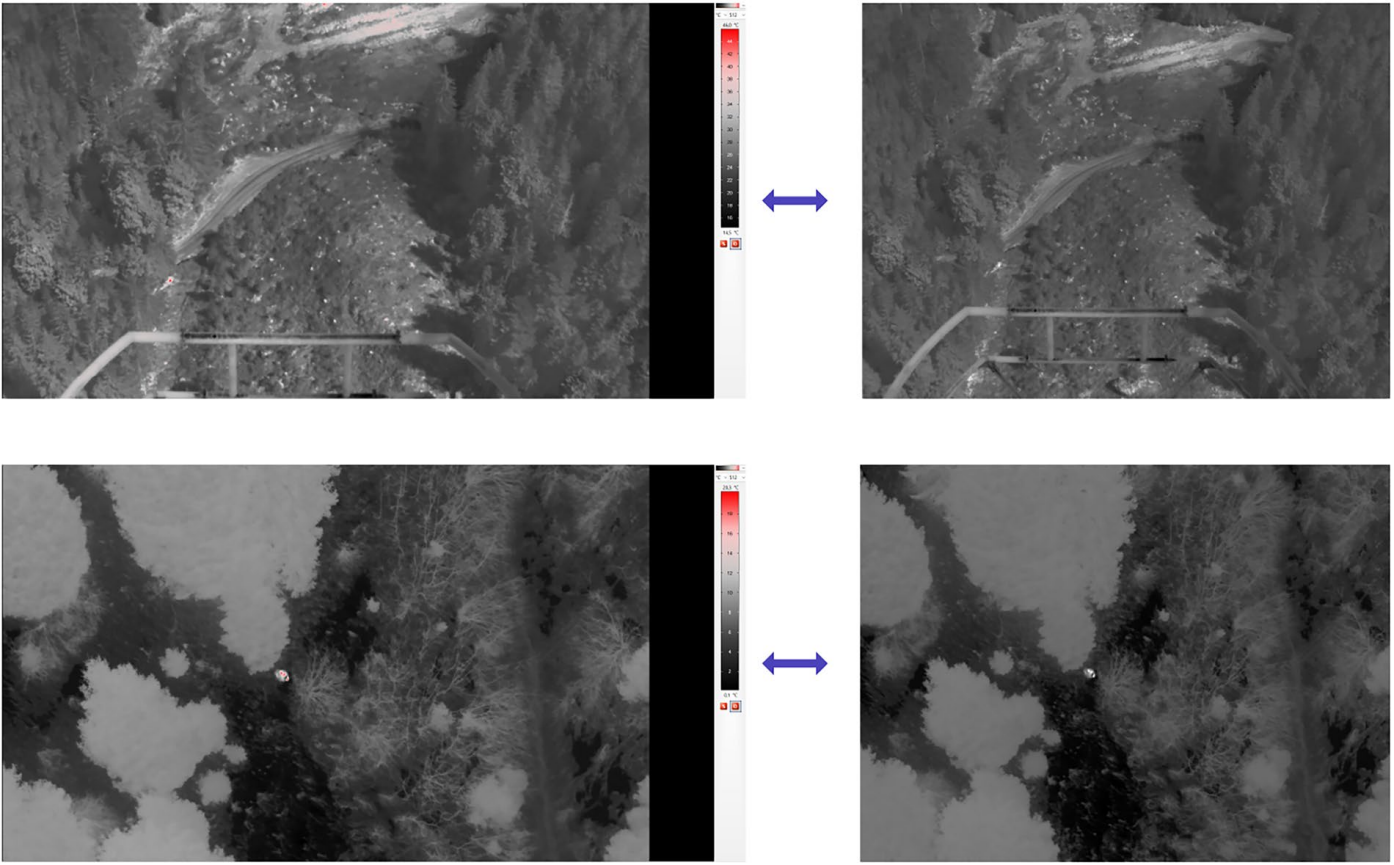
Exemplary frames showcasing the result of the data export process under different seasonal conditions. The upper images reflect a summer environment (range 14.5 ^°^C to 46 ^°^C) where human signatures often blend into the heated surroundings. The lower images show a high-contrast winter setting (range 0 ^°^C to 20 ^°^C). Each row compares the original thermal data including its scaling (left) with the resulting processed image (right).

InfraTec GmbH employs a proprietary data format for the storage of infrared images, known as the IRB-format. The initial procedure entailed the export of ASCII data from the infrared images. Following this step, the generation of images from the ASCII data was possible. In order to accomplish this objective, the highest and lowest temperature values in the image were first determined. Thereafter, the remaining temperature values were interpolated between the minimum and maximum values within the range of 0–255. Figure 4 presents the pre- and post-conversion images.

### Data Annotation

The dataset employed in this study consists of images containing human subjects under diverse environmental conditions. To enable supervised training of object detection models, each person within the images was manually annotated using bounding boxes. All annotations were performed using CVAT^37^ a dedicated web-based annotation platform, which provides a user-friendly interface for defining bounding boxes and supports a wide range of export formats (e.g., COCO JSON, Pascal VOC XML, and YOLO). Annotators were instructed to draw bounding boxes tightly around every visible person in an image, including partially occluded or truncated individuals. To ensure data quality and consistency, each annotated image underwent a two-step verification process. First, automated validation scripts were applied to detect structural or formatting errors. Second, manual review was conducted by an independent annotator to confirm the correctness of bounding box placement and label assignment. This dual-stage approach minimizes both systematic and human errors in the annotation process.

## Data Records

The AIResQ dataset contains high-resolution (up to 2048 × 1536 pixels) IR pictures with corresponding person labels and is available and can be accessed via the following https://zenodo.org/records/19677785, the benchmark dataset (additional information in Chapter Technical Validation) contains IR pictures in a resolution of 640 × 512 pixels with corresponding person labels and is freely available and is accessible via the following https://zenodo.org/records/17405074.

## Technical Validation

The validation of the datasets is comprised of two distinct yet complementary forms of validation: statistical and model based. The statistical validation process involves three distinct analyses. Firstly, an analysis of the overall distribution of the images throughout the year is conducted. Secondly, a distribution analysis of the number of bounding boxes is performed. Thirdly, a distribution analysis of the height of the bounding boxes in relation to the complete image is conducted. This analysis aims to ascertain the degree of realism with which the various datasets correspond to actual SAR mission data. The model based validation, on the other hand, is intended to determine the suitability of the datasets for the training of object detectors to detect persons on thermal images from SAR missions. A benchmark dataset has been compiled with the objective of establishing a realistic validation basis, which serves as the foundation for both types of validation.

### Data Distribution & Properties

The distribution of person labels is homogeneous across the dataset. As demonstrated in Table 2, the mean number of subjects observed per image is approximately two. This results in a wide variance in the number of people across the entire dataset.

**Table 2:** Statistical overview of the dataset distribution.

	Persons per Image [Mean]	Persons per Image [STD]	Persons Count
Total	1.79	2.11	17,550
High-Res	1.67	2.39	10,893
Med-Res	2.03	1.34	6,657
Winter	1.78	1.40	2,411
Spring	2.13	1.55	4,962
Summer	1.63	2.42	8,682
Autumn	1.93	2.14	1,495

The columns represent the mean number of persons per image, the standard deviation (STD), and the total person count. The rows provide a breakdown by image resolution (High-Resolution and Medium-Resolution) and meteorological seasons.The majority of person labels were collected during the spring and summer months, with a mean and standard deviation of approximately 2.0.

The recording days span a complete annual cycle in both resolutions (2048 × 1536 and 1024 × 768 pixels). The heightened recording activity occurs with greater frequency during the summer and spring months. Table 3 shows the annual distribution of the dataset and a higher recording frequency during summer and spring months, because the months of summer and spring present a particular challenge, as the contrast between the person and their surroundings is often minimal, rendering them more difficult to detect on thermal images.

**Table 3:** Distribution of images throughout the year: Heightened recording activity occurs with greater frequency during the summer and spring months.

	Winter	Spring	Summer	Autumn
Total	1358	2334	5320	776
2048 × 1536	740	2274	2816	676
1024 × 768	618	60	2504	100

### Benchmark Dataset

**Fig. 5:**
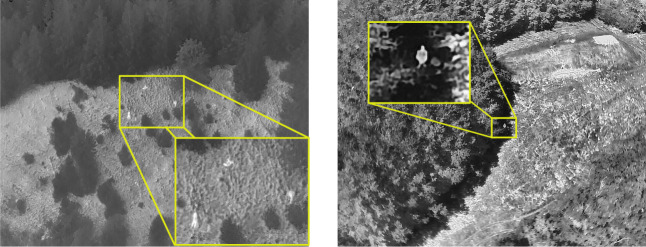
Example pictures of benchmark dataset of actual drone images in SAR missions. In the left picture in the center are three persons standing, sitting and lying. In the right picture one person sitting is highlighted.

The validation process utilizes a benchmark dataset for comparable validation purposes. The validation encompasses as metric the mean average precision (mAP) of several trained object detectors, each of which was trained with several datasets for comparison. The benchmark dataset comprises 1,988 images with 2,643 bounding boxes with a resolution of 640 × 512 pixel in a wilderness environment. Those images, as shown in Fig. 5, originate from real operational data of collaborating rescue organizations and police departments and UAV flights of joint exercises.

The validation approach is intended to test the practical suitability of the object detectors. The images of benchmark dataset were not used during the training, which is why the object detectors were validated after completing training sessions.

### Related Datasets

Most of the related datasets were collected via drone flights utilizing the same thermal cameras as those employed by SAR organizations during missions, during which individuals were recorded at a resolution of 640 × 512 pixels. The recording techniques employed in the datasets differ from capturing video streams and extracting frames to take pictures sets while flying. For example, the WiSARD dataset comprises individual images captured separately and the HIT-UAV dataset consists of individual frames extracted from disparate videos.

### Bounding Box Height Distribution

The investigated datasets diverge from the conventional deployment data typical of SAR missions. Firstly, the mean height of the bounding boxes in the benchmark dataset is markedly smaller. Secondly, the standard deviation of the height of the bounding boxes in the benchmark dataset is significantly lower than in the other datasets examined. Table 4 presents the number of bounding boxes in the various datasets, along with the mean and standard deviation of the height and width of the bounding boxes.

**Table 4:** Number of bounding boxes, mean sizes and standard deviation of the relative size of bounding boxes of the investigated datasets.

Dataset	Bounding Boxes	Mean Height	STD Height	Mean Width	STD Width
Benchmark	2,643	0.020	0.012	0.012	0.009
WiSARD	27,544	0.068	0.047	0.038	0.026
HIT-UAV	12.312	0.039	0.011	0.020	0.008
BirdsAI	12,531	0.075	0.044	0.023	0.009
RGBTDronePerson	60,258	0.027	0.006	0.015	0.005
NII-CU	23,037	0.046	0.029	0.016	0.009
POP	26,811	0.048	0.022	0.033	0.015
AIResQ (ours)	17,550	0.035	0.040	0.020	0.021

**Fig. 6:**
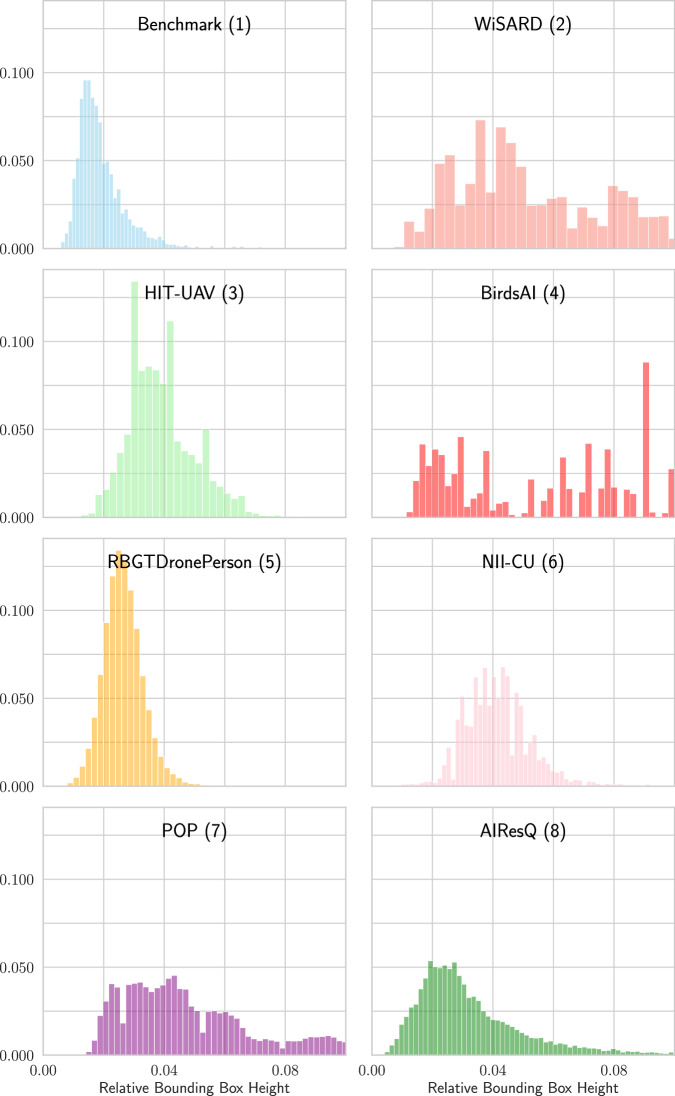
Distribution of bounding box heights across the various datasets under investigation. **1, 3, 5, 8**: Distribution of bounding box heights concentrates around 0.02 to 0.04 in relation to picture size and overall distribution is not widespread. The distribution of the benchmark dataset is the most realistic distribution in the context of SAR missions. **6, 7**: Distribution of bounding box heights is more widespread and does not concentrate on a specific value. **2, 3**: distribution of bounding box heights is found to be significantly more widespread and encompasses a substantially larger size in comparison to the benchmark dataset.

The distribution of the height of the bounding boxes is not normal distributed, so the absolute distribution of the height of the bounding boxes is a central metric for the assessment of datasets, serving to illustrate the prevalence of varying box heights within the overall image. If the distribution of bounding box heights aligns with that of the benchmark dataset, the dataset is deemed to reflect a realistic object size that corresponds to the reality of SAR missions. In order to create a practical and usable dataset for the training of object detectors (Chapter Accuracy Object Detection) for SAR missions, it is essential that the dimensions of the bounding boxes are as similar as possible to those of the real deployment data in the benchmark dataset. The distribution of the heights of the bounding boxes in the benchmark dataset, as show in Fig. 6, demonstrates a concentration of over 0.25 of heights within the range of 0.02, in relation to the overall picture. At a resolution of 640 × 512 pixels, over 25% of all pictures result in a total object height of approximately 10 pixels. The dispersion of the distribution is minimal in comparison to other datasets, as the WiSARD dataset, as illustrated in Fig. 6.

As shown in Table 4, the mean value of the bounding box height in the WiSARD dataset is approximately three times higher than in the benchmark dataset. This is also attributable to a wider distribution of heights, as evidenced in Fig. 6. There is no discernible concentration of heights, and the distribution is more widespread. The HIT-UAV and RGBTDrone datasets exhibit distributions that are comparable to that of the benchmark dataset. The majority of the variables are concentrated around 0.03, in comparison to the overall picture. Furthermore, the dispersion of the variables is markedly lower in comparison to the WiSARD dataset. Similar to the HIT-UAV and RGBTDrone dataset, the heights of bounding boxes in the AIResQ^34^ dataset are more concentrated around 0.02, with a less widespread distribution than in WiSARD. In comparison to the benchmark^35^ dataset, the distribution of heights in AIResQ^34^ is more widespread, with a greater range of larger bounding box heights.

In conclusion, the quantitative distribution of bounding box heights in AIResQ^34^ and HIT-UAV exhibits a greater degree of similarity to the benchmark dataset than other datasets such as WiSARD. The concentration of bounding boxes at a specific point is more pronounced, and the distribution is not as widespread.

### Accuracy Object Detection

People are detected using object detectors, which are special machine learning models designed to determine the position of objects and then classify them. Commonly used object detectors in SAR missions are YOLOv3^18^, YOLOv5^21^, Faster R-CNN or SSD^27^. A key metric for the accuracy of object detectors is the mAP50, which is described by Everingham *et al*.^38^. For validation purposes this study employs different models - YOLOv8^39^, YOLOv11^40^, Faster R-CNN^9^, RT-DETR^41^ and RetinaNET^10^ - which represents established architectures of the one- and two-stage and transformer models. For ensuring comparability of the training outcomes, all object detectors are trained with the same epoch size and hyperparameters with several datasets. The training of each object detector was conducted over the course of 200 epochs. The resulting accuracy of the object detectors is calculated by validating them on the benchmark dataset, as described in Chapter Benchmark Dataset.

**Fig. 7:**
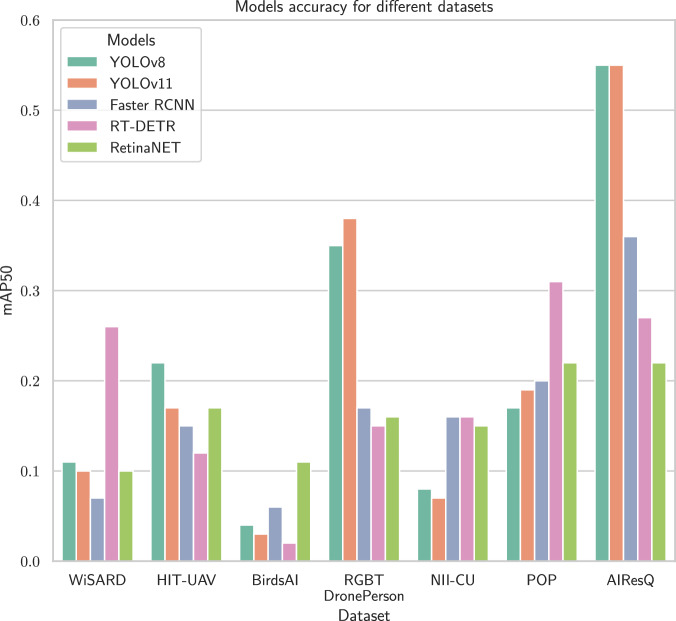
The training with the AIResQ dataset has been shown to result in a higher mAP50 in all of the investigated models, with the exception of RT-DETR and RetinaNET. The POP dataset has been identified as a reliable alternative for person detection on airborne infrared pictures in SAR missions in low resolution.

As illustrated in Fig. 7, the object detectors that were trained with the AIResQ^34^ dataset demonstrated a significantly higher accuracy compared to the object detectors that was trained with the related infrared datasets. However, the POP and WiSARD datasets constitute an exception to this, exhibiting a marginally elevated mAP50 for RT-DETR and RetinaNET. The YOLOv8 and YOLOv11 model, when trained with AIResQ^34^, achieved an mAP50 of 0.55 on the benchmark dataset, which is the highest mAP50 of all the trained models. In comparison, the RT-DETR model trained with the POP dataset demonstrated an mAP50 of 0.31 on the same benchmark dataset, which the highest score of model that is not trained with AIResQ^34^.

#### Human Comparison

The identification of individuals in infrared imagery captured from the perspective of an UAV represents a considerable cognitive challenge, even for emergency responders engaged in SAR operations. To the best of our knowledge, there have been no studies conducted on the efficacy of individuals in identifying others in infrared images of unknown subjects, nor on the decrease of people’s motivation and increase of mental fatigue by searching for tiny objects in pictures. Nevertheless, a substantial body of research has demonstrated that motivation levels decline and mental fatigue increases during extended periods of cognitive activity^42–44^. It is therefore the objective of this experiment to ascertain the comparative performance of trained and untrained individuals in relation to our object detector with the highest mAP50, with regard to the identification of people in low-resolution infrared images. In order to accomplish this objective, the benchmark dataset has been divided into ten clusters, with each cluster comprising 200 images that have been randomly selected.

Subsequently, the ten clusters were distributed randomly to five individuals with experience of IR images from a UAV perspective and five individuals with no experience. The experienced participants are members of SAR teams, which are familiar with the use of thermal imaging cameras on UAVs to locate missing persons, the inexperienced participants have not conducted a search on persons thermal images at any time. The participants were then tasked with the identification of locations within the imagery within ten seconds per picture in which the presence of a person was suspected, with this identification being made through the use of bounding boxes. A comparison was then drawn between the labels that were created with the ground truth labels in order to establish the precision and recall for this particular cluster:

$$Precision=\frac{TP}{TP+FP}$$

$$Recall=\frac{TP}{TP+FN}$$

**Fig. 8:**
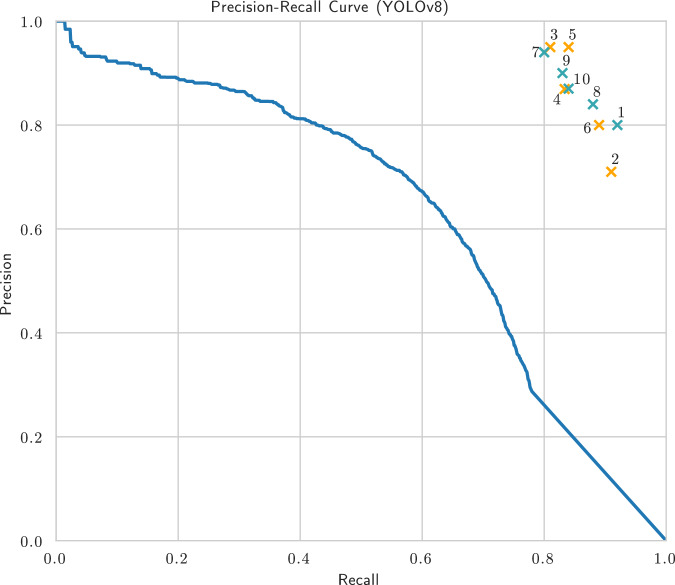
Precision-Recall curve of our best model (YOLOv8 (XL)) trained on AIResQ in comparison with human operators (cluster 1–10). Human operators showing in general a higher accuracy as our model, untrained participants (cluster 1, 7, 8, 9, 10; cyan mark) show a similar performance than trained participants (cluster 2, 3, 4, 5, 6; orange mark).

This was then juxtaposed with the mAP curve of the most effective model - as demonstrated in Fig. 8. All drawn bounding boxes were defined as TP if the overlap of the area with the ground truth was more than ten percent, since the mean value of the bounding box height in the benchmark dataset is only ten pixels. Subsequent to the delineation of the bounding boxes, the calculation of recall and precision for each cluster was conducted.

In general, all participants demonstrate higher levels of performance than the best-trained model, although the experienced participants only marginally exceed the inexperienced ones. In comparison to the model, which exhibited an approximate processing time of three milliseconds per image, the participants demonstrated a substantially slower rate of approximately ten seconds per image. Furthermore, it has been observed that individuals frequently experience a decline in concentration levels during professional activities, particularly when engaged in the analysis of extensive image sequences. Recent large-scale trials have demonstrated that mental fatigue acts as a performance bottleneck; it elevates the perceived exertion of physical tasks and compromises cognitive vigilance, even when physiological markers remain stable. This is particularly critical in mountain rescue, where cognitive load and physical demand coincide over extended periods^45^.

### Summary

In SAR missions the searched objectives are in majority around ten pixels in height which presents a significant challenge to the accuracy of object detection from models such as YOLOv8. AIResQ^34^ contains roughly 10,000 thermal images that improve the accuracy of thermal image specialized object detectors significantly on those small objectives. A YOLOv8 object detector trained with the AIResQ^34^ dataset demonstrated a significantly increase in accuracy compared to established datasets when evaluated on a real-life benchmark dataset in SAR mission context. This significant improvement in detection performance expands the possibilities of automated people search by enhancing the ability to locate small objects or people with greater precision. Further higher-resolution thermal cameras for drones are now available, with resolutions approaching that of AIResQ^34^. Consequently, the AIResQ^34^ data set has been designed to accommodate such resolutions.

### Ethics statement

Prior to the study, a formal legal data protection assessment was conducted by the independent law firm ByteLaw (Az: 08/23 DK01). This assessment confirmed that the infrared data collected is non-identifiable, as individuals cannot be distinguished based on body contours or thermal signatures. Participants were recruited voluntarily from the professional environment of the project partners in the guidelines of a university’s ethics review. The university’s ethics board confirmed this to us via a statement of ethical and data privacy approval. All participants provided informed oral consent for their data to be processed and for the results to be published in an anonymous format. Consequently, the study complies with all relevant data protection regulations, ensuring that the research findings do not allow for any re-identification of specific individuals.

## Data Availability

The AIResQ dataset (10.5281/zenodo.17405322) is available upon request via following https://zenodo.org/records/17405323. The AIResQ Benchmark dataset (10.5281/zenodo.17405073) is freely available via following https://zenodo.org/records/17405074.

## Code availability

The training scripts (based on Python) for object detection models are available via: https://github.com/Buettnej/DetectorSample (10.5281/zenodo.17651514). The repository contains the scripts used for YOLOv8, YOLO11, RetinaNet, Faster RCNN, and RT-DETR. There is a **project.toml** file that contains installation configurations. These configurations help create a virtual environment for training object detector models.
